# 3-[2-(1*H*-1,3-Benzodiazol-2-yl)eth­yl]-1,3-oxazolidin-2-one

**DOI:** 10.1107/S1600536811012256

**Published:** 2011-04-13

**Authors:** Giovanna Brancatelli, Francesco Nicoló, Sara De Grazia, Anna Maria Monforte, Alba Chimirri

**Affiliations:** a Dipartimento di Chimica Inorganica Chimica Analitica e Chimica Fisica, Universitá degli Studi di Messina, Via Salita Sperone 31, I-98166 Vill. S. Agata - Messina, Italy; b Dipartimento Farmaco-Chimico, Università di Messina, Viale Annunziata, 98168, Messina, Italy

## Abstract

In the title compound, C_12_H_13_N_3_O_2_, the dihedral angle between the oxazolone ring and the benzimidazole unit is 45.0 (5)°, exhibiting a staggered conformation at the Cα—Cβ bond. In the crystal, a strong N—H⋯N hydrogen bond links the mol­ecules into a *C*(4) chain along the *c* axis while a C—H⋯O hydrogen-bonding inter­action generates a *C*(5) chain along the *a* axis, *i.e.* perpendicular to the other chain.

## Related literature

For the therapeutic activity of benzimidazole and oxazolid­in­one derivatives, see: Niño *et al.* 2001 ▶; Siva Kumar *et al.* 2010 ▶; Zappia *et al.* 2007 ▶. For the drug linezolid [systematic name (*S*)-*N*-({3-[3-fluoro-4-(morpholin-4-yl)phen­yl]-2-oxo-1,3-oxazolidin-5-yl}meth­yl)acetamide], see: Brickner *et al.* (2008 ▶). For asymmetry of the exocyclic angles in oxazolone rings, see: Grassi *et al.* (2001 ▶). For the structures of benzimidazole and oxazolidine, see: Totsatitpaisan *et al.* (2008 ▶); Wouters *et al.* (1997 ▶).
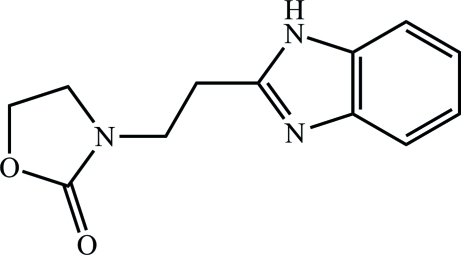


## Experimental

### 

#### Crystal data

C_12_H_13_N_3_O_2_
*M* *_r_* = 231.25
Monoclinic, 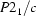

*a* = 6.0940 (2) Å
*b* = 18.1570 (6) Å
*c* = 10.0740 (3) Å
β = 90.696 (1)°
*V* = 1114.59 (6) Å^3^
*Z* = 4
Mo *K*α radiation
μ = 0.10 mm^−1^
*T* = 296 K
0.51 × 0.43 × 0.21 mm

#### Data collection

Bruker APEXII CCD diffractometer
34135 measured reflections
1951 independent reflections
1830 reflections with *I* > 2σ(*I*)
*R*_int_ = 0.021

#### Refinement

*R*[*F* ^2^ > 2σ(*F* ^2^)] = 0.033
*wR*(*F* ^2^) = 0.089
*S* = 1.05
1951 reflections
155 parameters
H-atom parameters constrained
Δρ_max_ = 0.21 e Å^−3^
Δρ_min_ = −0.22 e Å^−3^

### 

Data collection: *APEX2* (Bruker, 2007 ▶); cell refinement: *SAINT* (Bruker, 2007 ▶); data reduction: *SAINT*; program(s) used to solve structure: *SHELXS97* (Sheldrick, 2008 ▶); program(s) used to refine structure: *SHELXL97* (Sheldrick, 2008 ▶); molecular graphics: *SHELXTL* (Sheldrick, 2008 ▶); software used to prepare material for publication: *SHELXTL*.

## Supplementary Material

Crystal structure: contains datablocks global, I. DOI: 10.1107/S1600536811012256/bh2341sup1.cif

Structure factors: contains datablocks I. DOI: 10.1107/S1600536811012256/bh2341Isup2.hkl

Additional supplementary materials:  crystallographic information; 3D view; checkCIF report

## Figures and Tables

**Table 1:** Hydrogen-bond geometry (Å, °)

*D*—H⋯*A*	*D*—H	H⋯*A*	*D*⋯*A*	*D*—H⋯*A*
N1—H1⋯N8^i^	0.86	2.08	2.8959 (13)	158
C11—H11*A*⋯O14^ii^	0.97	2.53	3.2876 (16)	135

Symmetry codes: (i) 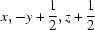; (ii) 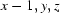.

**Fig. 1.**
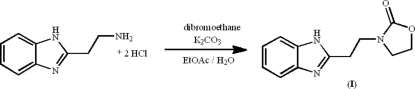
Synthesis reaction scheme of the title compound.

**Fig. 2.**
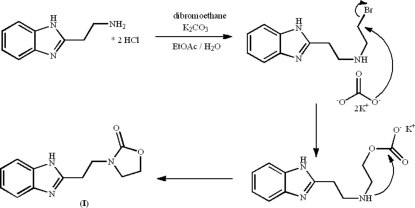
Mechanism proposed for the synthesis of the title compound.

**Fig. 3.**
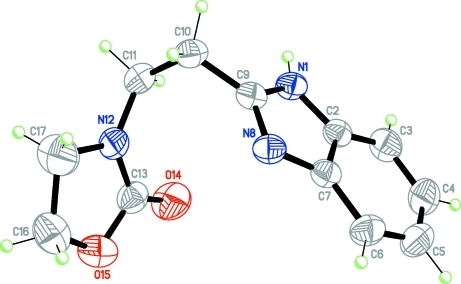
ORTEP drawing of the title molecule. Non H-atoms represented as displacement ellipsoids are plotted at the 50% probability level, while H atoms are shown as small spheres of arbitrary radius. In this view the staggered conformation around the Cα—Cβ bond is visible.

**Fig. 4.**
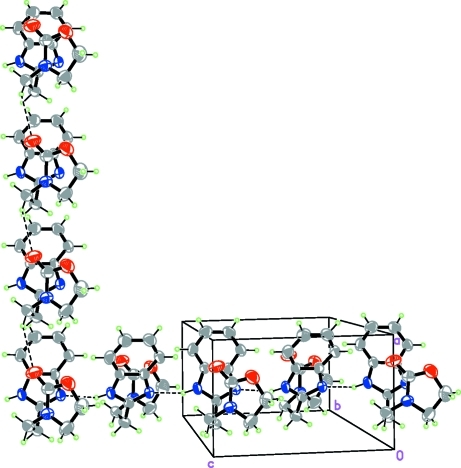
Arrangement of the molecules in perpendicular chains. The chain [C(5)] ruled by the C11—H11A···O14 interaction prolongs the crystal packing along the a axis; the other one [C(4)] generated by the N1—H1···N8 interaction is extended along the c axis. Dotted lines indicate H-bonding interactions.

## supplementary crystallographic information

### Comment

Heterocyclic compounds containing 5- or 6-membered rings are important for their diverse biological activities. In particular, the chemistry of oxazolidinone and benzimidazole has received considerable attention owing to their synthetic and biological importance.

Benzimidazole and oxazolidinone derivatives have been studied for the treatment of different pathologies. Their scaffold has been incorporated into a wide variety of therapeutically interesting compounds that show antibacterial, antifungal, antiviral, antineoplastics and cholinergic activity among others (Niño *et al.*, 2001; Siva Kumar *et al.*, 2010; Zappia *et al.*, 2007). Furthermore, the introduction in the pharmaceutical market of Linezolid, an oxazolidin-2-one-based antibacterial drug, attracted the interest of the scientists on this scaffold (Brickner *et al.*, 2008). On the basis of some common properties, such as antibacterial activity, of these two classes of heterocyclic compounds, in this study we synthesized the title molecule, in which the benzimidazole ring is linked to an oxazolidinone scaffold, with the aim to obtain a compound having two different moieties in the same molecular entity, and then a synergism of activity.

The one-pot synthetic route employed to obtain the title compound is depicted in Figure 1. Treatment of the commercially available 2-(2-aminoethyl)-benzimidazole dihydrochloride with dibromoethane and potassium carbonate gave the desired product. The proposed mechanism for the synthesis is shown in Figure 2. The nucleophilic attack of the 2-(2-aminoethyl)-benzimidazole primary amine function on the dibromoethane is followed by oxazolidinone ring formation. An excess of potassium carbonate is necessary both to create the basic medium for the *N*-alkylation and for the formation of the oxazolidinone moiety.

The molecule crystallizes in the centrosymmetric *P*2_1_/*c* space group. The asymmetric unit contains one molecule, shown in Figure 3. The dihedral angle between the oxazolone ring and the benzimidazole unit is 45.0 (5)°, exhibiting a staggered conformation at the Cα—Cβ bond. The carbonyl fragment displays pronounced asymmetry at the *exo*-cyclic angles, being N12—C13—O14 and O14—C13—O15 of 128.4 (1)° and 121.9 (1)°, respectively, because of both electronic and steric factors due to the presence of different atoms bound to C13 (Grassi *et al.*, 2001). The dimensions within the benzimidazole and the oxazolidine moieties are in excellent agreement with those found in the benzimidazole and oxazolidine crystal structures (Totsatitpaisan *et al.*, 2008; Wouters *et al.*, 1997).

Packing analysis of the crystal lattice indicates that the tridimensional molecular arrangement is ruled by many H-bonding interactions. A strong H-bond N1—H1···N8 gives rise to a molecular chain [C(4)] along the *c* axis (Figure 4). Another H-bonding interaction C11—H11···O14 generates a chain [C(5)] along the *a* axis, perpendicular to the previous one.

### Experimental

A solution of dibromoethane (3 mmol, 0.258 ml) in ethyl acetate (2 ml) was added over 10 minutes to a stirred mixture of 2-(2-aminoethyl)-benzimidazole dihydrochloride (1 mmol, 0.234 g), potassium carbonate (10 mmol, 1.38 g), ethyl acetate (5 ml) and water (2 ml). After the reaction mixture was refluxed for 36 h, the two phases were separated and the aqueous layer was extracted with ethyl acetate (2 x 5 ml). The combined organic phases were dried over anhydrous Na_2_SO_4_, filtered and concentrated. Elution with a mixture of chloroform and methanol (99:1) gave the title molecule as colourless crystals (yield: 30%). ^1^H-NMR (CDCl_3_, 300 MHz) δ 3.30 (t, *J* = 6.59, 2H, CH_2_), 3.61 (t, *J* = 6.71, 2H, CH_2_), 3.83 (t, *J* = 6.59, 2H, CH_2_), 4.28–4.34 (t, *J* = 6.71, 2H, CH_2_), 7.22–7.25 (m, 4H, Ar), 7.56 (bs, 1H, NH).

### Refinement

H atoms were located in a difference Fourier map and placed in idealized positions using the riding-model technique, with C—H = 0.93 and 0.97Å for aromatic H and methylene H, respectively, and N—H = 0.86Å, and with *U*_iso_(H) = 1.2*U*_eq_(C, N).

### Crystal data

**Table:**

C_12_H_13_N_3_O_2_	*F*(000) = 488
*M**_r_* = 231.25	*D*_x_ = 1.378 Mg m^−^^3^
Monoclinic, *P*2_1_/*c*	Mo *K*α radiation, λ = 0.71073 Å
Hall symbol: -P 2ybc	Cell parameters from 9799 reflections
*a* = 6.0940 (2) Å	θ = 2.3–30.0°
*b* = 18.1570 (6) Å	µ = 0.10 mm^−^^1^
*c* = 10.0740 (3) Å	*T* = 296 K
β = 90.696 (1)°	Prism, colourless
*V* = 1114.59 (6) Å^3^	0.51 × 0.43 × 0.21 mm
*Z* = 4	

### Data collection

**Table:**

Bruker APEXII CCD diffractometer	*R*_int_ = 0.021
graphite	θ_max_ = 25°, θ_min_ = 3.0°
φ and ω scans	*h* = −7→7
34135 measured reflections	*k* = −21→21
1951 independent reflections	*l* = −11→11
1830 reflections with *I* > 2σ(*I*)	

### Refinement

**Table:**

Refinement on *F*^2^	Secondary atom site location: difference Fourier map
Least-squares matrix: full	Hydrogen site location: inferred from neighbouring sites
*R*[*F*^2^ > 2σ(*F*^2^)] = 0.033	H-atom parameters constrained
*wR*(*F*^2^) = 0.089	*w* = 1/[σ^2^(*F*_o_^2^) + (0.0448*P*)^2^ + 0.2758*P*] where *P* = (*F*_o_^2^ + 2*F*_c_^2^)/3
*S* = 1.05	(Δ/σ)_max_ < 0.001
1951 reflections	Δρ_max_ = 0.21 e Å^−^^3^
155 parameters	Δρ_min_ = −0.22 e Å^−^^3^
0 restraints	Extinction correction: *SHELXL97* (Sheldrick, 2008)
0 constraints	Extinction coefficient: 0.031 (3)
Primary atom site location: structure-invariant direct methods	

### Fractional atomic coordinates and isotropic or equivalent isotropic displacement parameters (Å^2^)

**Table:**

	*x*	*y*	*z*	*U*_iso_*/*U*_eq_	
C2	0.68193 (19)	0.18737 (6)	0.48419 (11)	0.0360 (3)	
C3	0.8441 (2)	0.14478 (8)	0.54393 (13)	0.0495 (3)	
H3	0.8494	0.1375	0.6353	0.059*	
C4	0.9971 (2)	0.11369 (8)	0.46129 (15)	0.0556 (4)	
H4	1.1078	0.0845	0.4978	0.067*	
C5	0.9903 (2)	0.12476 (8)	0.32465 (15)	0.0523 (4)	
H5	1.0971	0.1032	0.2722	0.063*	
C6	0.8292 (2)	0.16686 (7)	0.26555 (12)	0.0452 (3)	
H6	0.825	0.1739	0.1741	0.054*	
C7	0.67213 (19)	0.19869 (6)	0.34697 (11)	0.0350 (3)	
C9	0.40064 (19)	0.25761 (6)	0.42951 (11)	0.0352 (3)	
C10	0.2094 (2)	0.30701 (7)	0.44963 (13)	0.0439 (3)	
H10A	0.1152	0.286	0.5167	0.053*	
H10B	0.1252	0.3104	0.3675	0.053*	
C11	0.2802 (2)	0.38413 (7)	0.49276 (13)	0.0439 (3)	
H11A	0.1517	0.4119	0.5187	0.053*	
H11B	0.3766	0.3802	0.5698	0.053*	
C13	0.6099 (2)	0.43147 (7)	0.38402 (14)	0.0457 (3)	
C16	0.4669 (3)	0.49106 (10)	0.20459 (17)	0.0682 (5)	
H16A	0.4673	0.4673	0.1184	0.082*	
H16B	0.455	0.5439	0.1917	0.082*	
C17	0.2788 (3)	0.46309 (11)	0.28639 (17)	0.0693 (5)	
H17A	0.193	0.5033	0.3221	0.083*	
H17B	0.1835	0.4308	0.235	0.083*	
N1	0.50576 (16)	0.22534 (6)	0.53358 (9)	0.0379 (3)	
H1	0.4689	0.2281	0.6156	0.045*	
N8	0.49276 (16)	0.24297 (6)	0.31510 (9)	0.0376 (3)	
N12	0.39262 (16)	0.42375 (6)	0.38915 (11)	0.0440 (3)	
O14	0.74742 (17)	0.40717 (7)	0.45882 (13)	0.0730 (4)	
O15	0.66295 (17)	0.47348 (6)	0.27768 (11)	0.0606 (3)	

### Atomic displacement parameters (Å^2^)

**Table:**

	*U*^11^	*U*^22^	*U*^33^	*U*^12^	*U*^13^	*U*^23^
C2	0.0400 (6)	0.0367 (6)	0.0314 (6)	−0.0057 (5)	0.0019 (5)	−0.0004 (5)
C3	0.0553 (8)	0.0536 (8)	0.0394 (7)	0.0006 (6)	−0.0045 (6)	0.0092 (6)
C4	0.0496 (8)	0.0541 (8)	0.0630 (9)	0.0100 (6)	−0.0035 (7)	0.0054 (7)
C5	0.0466 (7)	0.0547 (8)	0.0558 (8)	0.0051 (6)	0.0086 (6)	−0.0087 (6)
C6	0.0471 (7)	0.0533 (8)	0.0352 (6)	−0.0018 (6)	0.0067 (5)	−0.0050 (5)
C7	0.0379 (6)	0.0370 (6)	0.0300 (6)	−0.0057 (5)	0.0017 (5)	−0.0015 (4)
C9	0.0360 (6)	0.0383 (6)	0.0315 (6)	−0.0071 (5)	0.0025 (5)	−0.0013 (5)
C10	0.0346 (6)	0.0494 (7)	0.0478 (7)	−0.0031 (5)	0.0067 (5)	−0.0006 (6)
C11	0.0395 (7)	0.0483 (7)	0.0440 (7)	0.0040 (5)	0.0050 (5)	−0.0045 (5)
C13	0.0392 (7)	0.0457 (7)	0.0521 (7)	−0.0010 (5)	0.0014 (6)	−0.0063 (6)
C16	0.0762 (11)	0.0666 (10)	0.0618 (10)	0.0016 (8)	0.0012 (8)	0.0156 (8)
C17	0.0548 (9)	0.0894 (12)	0.0634 (10)	−0.0038 (8)	−0.0136 (7)	0.0212 (9)
N1	0.0433 (6)	0.0459 (6)	0.0246 (5)	−0.0031 (4)	0.0056 (4)	−0.0011 (4)
N8	0.0386 (5)	0.0459 (6)	0.0283 (5)	−0.0023 (4)	0.0020 (4)	0.0011 (4)
N12	0.0341 (5)	0.0451 (6)	0.0527 (6)	0.0013 (4)	−0.0023 (5)	0.0048 (5)
O14	0.0389 (6)	0.0963 (9)	0.0835 (8)	0.0045 (5)	−0.0103 (5)	0.0128 (7)
O15	0.0534 (6)	0.0681 (7)	0.0605 (6)	−0.0114 (5)	0.0104 (5)	0.0016 (5)

### Geometric parameters (Å, °)

**Table:**

C2—N1	1.374 (2)	C10—H10A	0.97
C2—C3	1.387 (2)	C10—H10B	0.97
C2—C7	1.398 (2)	C11—N12	1.447 (2)
C3—C4	1.378 (2)	C11—H11A	0.97
C3—H3	0.93	C11—H11B	0.97
C4—C5	1.391 (2)	C13—O14	1.204 (2)
C4—H4	0.93	C13—N12	1.333 (2)
C5—C6	1.374 (2)	C13—O15	1.357 (2)
C5—H5	0.93	C16—O15	1.432 (2)
C6—C7	1.3934 (17)	C16—C17	1.508 (2)
C6—H6	0.93	C16—H16A	0.97
C7—N8	1.391 (2)	C16—H16B	0.97
C9—N8	1.315 (2)	C17—N12	1.430 (2)
C9—N1	1.355 (2)	C17—H17A	0.97
C9—C10	1.487 (2)	C17—H17B	0.97
C10—C11	1.5269 (19)	N1—H1	0.86
			
N1—C2—C3	132.72 (11)	N12—C11—H11A	109.1
N1—C2—C7	105.08 (10)	C10—C11—H11A	109.1
C3—C2—C7	122.20 (12)	N12—C11—H11B	109.1
C4—C3—C2	116.75 (12)	C10—C11—H11B	109.1
C4—C3—H3	121.6	H11A—C11—H11B	107.8
C2—C3—H3	121.6	O14—C13—N12	128.4 (1)
C3—C4—C5	121.75 (13)	O14—C13—O15	121.9 (1)
C3—C4—H4	119.1	N12—C13—O15	109.6 (1)
C5—C4—H4	119.1	O15—C16—C17	106.2 (1)
C6—C5—C4	121.43 (13)	O15—C16—H16A	110.5
C6—C5—H5	119.3	C17—C16—H16A	110.5
C4—C5—H5	119.3	O15—C16—H16B	110.5
C5—C6—C7	117.89 (12)	C17—C16—H16B	110.5
C5—C6—H6	121.1	H16A—C16—H16B	108.7
C7—C6—H6	121.1	N12—C17—C16	101.45 (13)
N8—C7—C6	130.32 (11)	N12—C17—H17A	111.5
N8—C7—C2	109.70 (10)	C16—C17—H17A	111.5
C6—C7—C2	119.98 (11)	N12—C17—H17B	111.5
N8—C9—N1	112.8 (1)	C16—C17—H17B	111.5
N8—C9—C10	125.8 (1)	H17A—C17—H17B	109.3
N1—C9—C10	121.3 (1)	C9—N1—C2	107.50 (9)
C9—C10—C11	111.88 (10)	C9—N1—H1	126.2
C9—C10—H10A	109.2	C2—N1—H1	126.2
C11—C10—H10A	109.2	C9—N8—C7	104.90 (10)
C9—C10—H10B	109.2	C13—N12—C17	113.11 (12)
C11—C10—H10B	109.2	C13—N12—C11	124.01 (11)
H10A—C10—H10B	107.9	C17—N12—C11	122.74 (11)
N12—C11—C10	112.67 (10)	C13—O15—C16	109.01 (11)
			
N1—C2—C3—C4	179.30 (13)	C3—C2—N1—C9	−179.94 (13)
C7—C2—C3—C4	−0.01 (19)	C7—C2—N1—C9	−0.55 (12)
C2—C3—C4—C5	0.4 (2)	N1—C9—N8—C7	−0.69 (13)
C3—C4—C5—C6	−0.5 (2)	C10—C9—N8—C7	176.10 (11)
C4—C5—C6—C7	0.3 (2)	C6—C7—N8—C9	−179.88 (12)
C5—C6—C7—N8	−179.73 (12)	C2—C7—N8—C9	0.32 (13)
C5—C6—C7—C2	0.05 (18)	O14—C13—N12—C17	177.42 (16)
N1—C2—C7—N8	0.15 (13)	O15—C13—N12—C17	−2.03 (17)
C3—C2—C7—N8	179.62 (11)	O14—C13—N12—C11	1.5 (2)
N1—C2—C7—C6	−179.68 (11)	O15—C13—N12—C11	−177.92 (11)
C3—C2—C7—C6	−0.20 (18)	C16—C17—N12—C13	5.96 (19)
N8—C9—C10—C11	−97.20 (14)	C16—C17—N12—C11	−178.09 (13)
N1—C9—C10—C11	79.34 (14)	C10—C11—N12—C13	−101.12 (15)
C9—C10—C11—N12	68.0 (1)	C10—C11—N12—C17	83.38 (16)
O15—C16—C17—N12	−7.44 (18)	O14—C13—O15—C16	177.27 (14)
N8—C9—N1—C2	0.81 (13)	N12—C13—O15—C16	−3.24 (16)
C10—C9—N1—C2	−176.15 (10)	C17—C16—O15—C13	6.84 (18)

### Hydrogen-bond geometry (Å, °)

**Table:**

*D*—H···*A*	*D*—H	H···*A*	*D*···*A*	*D*—H···*A*
N1—H1···N8^i^	0.86	2.08	2.8959 (13)	158.
C11—H11A···O14^ii^	0.97	2.53	3.2876 (16)	135.
C11—H11B···O14	0.97	2.58	2.9019 (16)	100.
C3—H3···O15^i^	0.93	2.73	3.3820 (17)	128.
C5—H5···O15^iii^	0.93	2.82	3.6229 (17)	145.
C6—H6···O14^iv^	0.93	2.66	3.4008 (17)	137.

Symmetry codes: (i) *x*, −*y*+1/2, *z*+1/2; (ii) *x*−1, *y*, *z*; (iii) −*x*+2, *y*−1/2, −*z*+1/2; (iv) *x*, −*y*+1/2, *z*−1/2.
